# Droitwich and Its Brine Baths

**Published:** 1888-03

**Authors:** Alfred Crespi

**Affiliations:** Wimborne, Dorset


### DROITWICH AND ITS BRINE BATHS.

BY

ALFRED CRESPI, M.R.C.S.,

*Wimborne, Dorset.*

Medical fashions are always changing. When I was a child, there was nothing like Torquay, Ventnor, and Penzance for consumptive invalids who could not get to Nice, Mentone, or, still better, to Madeira. Now we have altered all that. Instead of advising patients to seek a comparatively mild but rather humid atmosphere, we send them—that is to say, the almost incalculably small fraction who can afford such a costly journey—to the sunny and rainless uplands of Colorado, the bracing highlands of Minnesota, and the snow-covered valleys of Davos and the Engadine. Had we in England lofty plains or sheltered valleys, 5,000 feet above sea-level, no doubt they would soon be crowded with invalids. Imagination—

that potent factor in the cure of disease—has much to do with the result. Who has not heard of the sufferer for whom his physician prescribed, without benefit, nitrate of potash, and who, some time later, after he had dismissed his doctor, met the latter, and, with a smiling countenance, announced himself cured. "What," asked the physician, "cured you?" "Why, to be sure, some saltpetre, which a friend, who had also had rheumatism badly, urged me to take regularly for three months." The very remedy prescribed by his physician, but taken with more faith and under a different name, had cured him. Change of air—implying, as it generally does, change of food and water, complete rest, fresh faces, and modified habits—is invaluable. Who has not felt that a fresh church, unfamiliar streets, unaccustomed voices, even a strange doctor, into whose sympathetic ear to pour one's complaints, are powerful factors in effecting a cure? Davos, Colorado Springs, and Orotave will have their day, as Florida, Falmouth, Malta, and Sea voyages have had theirs; and then we shall send patients to find health amid still more unfamiliar surroundings—for example, for a few weeks in Tierra del Fuego, or for a trip to Mount Erebus, or in a journey across Siberia in January, or in a residence in a tent in the Sahara. The fashionable physician can never be at a loss for some place with manifold and untried advantages to which to recommend his wealthy clients.

Droitwich has lately been coming prominently into notice. It has no extensive forests near, no dazzling Russian or Spanish sky, no mild humidity, no furnace-like heat and dryness to recommend it. Its sole claim rests upon its Brine Baths, and upon their efficacy, tested by thousands of patients, in gout and chronic rheumatism.

For nearly two thousand years brine has been pumped up in enormous quantities, evaporated, and made into salt of excellent quality. This has led to Droitwich becoming well known far and near, as well as to certain curious sinkings of the land, interesting to the visitor, but not pleasant and reassuring to the householder. The brine springs of Droitwich are remarkable for their great purity and strength, containing as they do nearly, if not quite, 50 per cent. of salt. Fifty years ago, attention was first drawn to the value of hot brine baths as therapeutic agents; and the late Mr. Bainbrigge, an excellent and experienced surgeon of the town, did much to push the place. The first pioneer in such an undertaking is rarely rewarded, and Mr. Bainbrigge went his way, having done less good to himself than he deserved. His son, Dr. W. P. Bainbrigge, is following in his father's steps, and paying particular attention to the properties of the baths, and to the cases in which they are most beneficial. It is his opinion, and it was confirmed by patients whom I carefully examined and questioned, that a course of these baths is invaluable in many of those cases of gout and rheumatism unfortunately so common in England, more particularly among the elderly poor who have had years of exposure to the weather, and among the well-to-do who have inherited some tendency to gout, or have long led a sedentary and self-indulgent life. From the great strength and coldness of the brine, it is necessary to dilute it with an equal quantity of hot water. After a time, the swollen and tender joints become less sensitive, and return more or less completely to their natural size, while the skin gets soft and velvety. Of late, accommodation on a large and luxurious scale has been provided for the increasingly large number of sufferers flocking to the

2 *

town. There are several private boarding-houses, which afford, at moderate charges, every convenience and attention; and two large hotels—the Raven, an extensive and handsome building, with delightful grounds, comfortable rooms, and cheerful surroundings; and the Royal. The latter I have not had an opportunity of going over, though the accounts I have received are most satisfactory. Mr. John Corbett, M.P. for the district, has done much for the place, and has developed the salt industry. He has also built some extensive baths, fitted up with the latest and most perfect appliances for treatment and comfort. When I was last at Droitwich, in September, I was much pleased with Mr. Corbett's arrangements, and the accommodation seemed likely to suffice for years; but I have just heard, I must confess, with unmingled surprise, that the new baths are no longer large enough to accommodate the many visitors whom the growing fame of Droitwich is attracting; and very considerable additions to them are now being made. To prevent disappointment, I must add that Mr. Corbett's Brine Baths are not a hotel; patients must stay elsewhere, going to the baths once or twice a day: though Droitwich is so small, that there is no difficulty in managing that.

To get the full benefit of the treatment, patients should stay some time. It is becoming increasingly the fashion for invalids to rush from place to place, stopping here for a week, and somewhere else for three days. Still, I was rather startled, on talking with a civil engineer—whose fingers showed that he was not cured, though he admitted that his brief stay had done him great good—to find that he proposed, on the following day, going to St. Leonard's, and a few days later setting off for Vienna, returning home by the Rhine.

Many persons who have tried Droitwich once, have found so much good that they have returned again and again, though it can rarely happen that these baths—or, indeed, any others—can eradicate the tendency to a disease as constitutional as gout, and, while curing the patient, make him proof for the future against a relapse. There is a general agreement that, with some exceptions, conspicuous good is done. Scrupulous attention to diet, and leaving off spices, stimulants, and meat, would keep at bay the old enemy; but patients seldom care to keep themselves well, though anxious enough to be cured. Some years ago I cautioned a Lancashire vicar against bad habits, and urged him to take active exercise every day, to discontinue wine and smoking, and to leave off late and luxurious dinners—and, in short, to live naturally and temperately. Without hesitation, he emphatically declared that he would rather have gout every day of his life than carry out my directions. I took my leave, wondering how he had the audacity to preach self-sacrifice, and to hold up the example of John the Baptist, Elijah, and the Master to his flock, while his own life reflected so little of the spirit of their teaching! Another friend of mine, also a very self-indulgent clergyman, who had tried all the baths he could think of, and had suffered many things at the hands of his physicians, and was none the better, but rather the worse, at last, at fifty-five, completely changed his habits, restricting himself to small quantities of plain food and simple fruits, and giving up at the same time stimulants, meat, and tobacco. The result was marvellous: he became young again, found himself able to walk and run with the most active, and, without visiting any more baths at home or abroad, lived for years a healthy and painless existence. He became a bitter—

indeed, a vindictive—foe of the doctors, crediting them with causing and encouraging illness for selfish ends; though it is likely enough that, up to the time of his conversion to simple habits, he had treated with supreme contempt the advice given him by his more far-seeing physicians. "Let us lead vicious and unnatural lives, and you must be good enough to make us well," is the demand of too many people of their doctor.

Brine baths and change are very well, so is good medical supervision; but, to get the full benefit they are capable of affording, attention to diet is not less important.

Droitwich is not in itself very attractive, nor is the country in the immediate neighbourhood remarkable, though pretty and fertile enough. Worcester, with its beautiful cathedral, is only six miles off; and Malvern, with its charming hills and pretty streets, is only fourteen miles away; while the luxuriant district of which Ledbury is the centre is barely twenty miles off. While one would not willingly choose Droitwich as a permanent residence, it is not worse than many other manufacturing towns, and decidedly better than some others. Its small size—the population is only 4,000, and even this includes a large area—enables the visitor to run off into the country at once: a quarter of an hour would be enough to get out into the fields and lanes; while a few minutes in the train will take him into the loveliest parts of Worcestershire. After all, the first consideration is to get well; and the comfort of feeling that he is getting good would reconcile a man to longer journeys and less attractive places. There is some fine timber close at hand, the Lickeys can be reached in half an hour, and some of the drives are charming and leave nothing to desire.

A great future lies before Droitwich as a health resort, and a more brilliant future before its staple industry there could hardly be. It is a question whether, as far as its baths go, it ranks as high as it ought. Hereford and Birmingham now send contingents of patients, who return greatly benefited ; while London is learning that it is not always necessary to wander off to Germany, while Droitwich is so near. I cannot speak positively as to the expenses of a residence at Droitwich, but should not imagine them to be great; and there is no reason to believe that those most deeply interested in the prosperity of the town will follow the bad example of some other health resorts, and frighten away visitors by exorbitant charges.

